# Novel Diagnostic and Therapeutic Methods in Ocular Surface and Corneal Diseases

**DOI:** 10.1155/2017/6137821

**Published:** 2017-04-16

**Authors:** Anna K. Nowinska, László Módis, Robert Koprowski, Jesús Merayo-Lloves

**Affiliations:** ^1^Clinical Department of Ophthalmology, School of Medicine with the Division of Dentistry in Zabrze, Medical University of Silesia, Katowice, Poland; ^2^Department of Ophthalmology, University of Debrecen, Debrecen, Hungary; ^3^Department of Biomedical Computer Systems, Faculty of Computer Science and Materials Science, Institute of Computer Science, University of Silesia, Sosnowiec, Poland; ^4^Instituto Oftalmológico Fernández-Vega, Oviedo, Spain

Noninfectious, chronic ocular surface and corneal diseases involve disorders in which structural changes are caused by various pathomechanisms including, but not limited to, inflammation, degeneration, autoimmunization, or dystrophy.

In the last 10 years, there has been a huge progress in our understanding of corneal disorders due to the availability of new in vivo imaging techniques, such as confocal microscopy, optical coherence tomography, corneal topography, or aberrometry. Recent advances in treatment methods include new cellular and medical, topical therapy with new biotechnology agents and novel treatment methods of keratoconus as well as further improvement of keratoplasty procedures.

Authors contributed a range of papers including 2 original research on novel anterior eye segment diagnostic methods and 5 original research on innovative ocular surface therapies.

A brief description of these 7 works is detailed below.

Novel diagnostic methods are the following.

The paper authored by R. Koprowski and L. Tian proves that blood pulsation has a statistically significant effect on the results of intraocular pressure measurement performed by Corvis Scheimpflug tonometer. The range of changes in the intraocular pressure measurement is high and can vary up to ±2.31 mmHg. For this reason, authors propose that, in modern ophthalmic devices, the measurement should be synchronized with the heartbeat phases.

The study “Keraring Intrastromal Segment Depth Measured by Spectral-Domain Optical Coherence Tomography in Eyes with Keratoconus” addresses a clinical problem of keratoconus surgery and intrastromal corneal ring segment (ICRS) implantation. The agreement between measured and intended distance of ICRS from the anterior and posterior corneal surfaces was evaluated. The authors proposed a method to analyze the implantation depth of the ICRS in the corneal stroma by measuring the distance of the inner corner from the posterior corneal surface which showed the best agreement with the intended distance.

Novel therapeutic methods are the following.

K. Krysik et al. reported their results on surgical treatment of corneal perforations. The authors analyzed a study group of 247 eyes with corneal perforation with a 6-year observation period. The three surgical procedures, dependent on size and location of perforation, were performed: full-sized penetrating keratoplasty, corneoscleral patch graft, and anterior lamellar keratoplasty. Although a complex treatment approach, the complication rates observed by authors were very high: graft melting was reported in 45 eyes (18.2%), glaucoma was diagnosed in 69 eyes (28%), and reinfections were reported in a total of 34 eyes (13.7%). Based on their observation, the authors claim that the therapeutic approach to corneal perforation is challenging and unpredictable. The further development of surgical and lamellar surgical techniques applied in corneal perforations is also necessary.

In the paper titled “A Novel Technique for Conjunctivoplasty in a Rabbit Model: Platelet-Rich Fibrin (RRF) Membrane Grafting” by M. E. Can et al., the effect of platelet-rich fibrin membrane (PRFM) on wound healing in experimental conjunctival tissue damage was investigated. PRF membrane was proved to have the beneficial effects on conjunctival healing. Besides its chemical effects, it provides mechanical support as a scaffold for the migrating cells that are important for the ocular surface regeneration. Authors promising results on PRF membrane application could encourage surgeons to applicate autologous PRF membrane as a growth factor-enriched endogenous scaffold for ocular surface reconstruction.

The authors of the paper “Biocompatibility and Biomechanical Effect of Single Wall Carbon Nanotubes Implanted in the Corneal Stroma: A Proof of Concept Investigation” conducted an experimental investigation where New Zealand rabbits were treated with a composition of carbon nanotubes (CNTs) suspended in balanced saline solution which was applied in the corneal tissue. Biocompatibility of CNTs has been previously reported in other fields of biology and medicine, for example, tissue engineering, regenerative medicine, drug delivery systems, and reinforcement of biological tissues. In the present study, it was found that carbon nanomaterials do not induce any inflammatory or foreign body reaction in the corneal stroma. The corneal biomechanical evaluation, as performed in this investigation, showed that there is a trend to obtain more rigidity of the corneal tissue after carbon nanostructure implantation, although these changes were not statistically significant. This method of treatment seems to be very promising in the variety of ectatic corneal disorders, but further research is necessary in order to comprehend and improve the biomechanical assessment of the present investigation and also to understand the potential use of these materials and this novel technology for the development of new applications of nanomedicine in visual sciences.

The purpose of the retrospective study “Active Pedicle Epithelial Flap Transposition Combined with Amniotic Membrane Transplantation for Treatment of Nonhealing Corneal Ulcers” was to evaluate the efficacy of active pedicle epithelial flap transposition combined with amniotic membrane transplantation in treating persistent corneal ulcer with epithelial nonhealing. The authors stated that the active epithelial flap inhibited inflammatory cell infiltration in the inflamed tissue and reduced the quantity of proteinases and cytokines released into the inflammatory cornea. Covering the ulcer with the active epithelial flap provided a relatively healthy substrate and microenvironment. This facilitated epithelial migration, reinforced basal epithelial adhesion, and promoted ocular surface healing. The proposed method has limitations, because this combined surgery is not suitable for corneal ulcers with a diameter exceeding 5 mm which are hard to make a peripheral epithelial flap to cover it. Despite its limitations, it seems a promising treatment method of the management of nonhealing corneal ulcers, which is one of the most difficult challenges faced by ophthalmologists.

Authors of the paper “Evaluation of Monocular Treatment for Meibomian Gland Dysfunction with an Automated Thermodynamic System in Elderly Chinese Patients: A Contralateral Eye Study” present a prospective, examiner-masked, contralateral eye clinical trial on the safety and efficacy of monocular treatment for elderly Chinese patients with meibomian gland dysfunction (MGD) with an automated thermodynamic system (LipiFLow). All patients had a significant reduction in dry eye symptoms accompanied by an increase of invasive tear breakup time (ITBUT) and meibomian glands yielding liquid secretion (MGYLS) and a reduction in corneal staining compared with the baseline parameters. LipiFlow was proved to be an effective treatment option for MGD.

